# An early Oligocene fossil demonstrates treeshrews are slowly evolving “living fossils”

**DOI:** 10.1038/srep18627

**Published:** 2016-01-14

**Authors:** Qiang Li, Xijun Ni

**Affiliations:** 1 Key Laboratory of Vertebrate Evolution and Human Origins, Institute of Vertebrate Palaeontology and Palaeoanthropology, Chinese Academy of Sciences, 142 Xi Zhi Men Wai Street, Beijing, 100044, China; 2 CAS Center for Excellence in Tibetan Plateau Earth Sciences, Beijing, 100101, China

## Abstract

Treeshrews are widely considered a “living model” of an ancestral primate, and have long been called “living fossils”. Actual fossils of treeshrews, however, are extremely rare. We report a new fossil species of *Ptilocercus* treeshrew recovered from the early Oligocene (~34 Ma) of China that represents the oldest definitive fossil record of the crown group of treeshrews and nearly doubles the temporal length of their fossil record. The fossil species is strikingly similar to the living *Ptilocercus lowii*, a species generally recognized as the most plesiomorphic extant treeshrew. It demonstrates that *Ptilocercus* treeshrews have undergone little evolutionary change in their morphology since the early Oligocene. Morphological comparisons and phylogenetic analysis support the long-standing idea that *Ptilocercus* treeshrews are morphologically conservative and have probably retained many characters present in the common stock that gave rise to archontans, which include primates, flying lemurs, plesiadapiforms and treeshrews. This discovery provides an exceptional example of slow morphological evolution in a mammalian group over a period of 34 million years. The persistent and stable tropical environment in Southeast Asia through the Cenozoic likely played a critical role in the survival of such a morphologically conservative lineage.

“Living fossils” are those groups where extant species and their ancient fossil relatives exhibit great morphological similarity as the result of slow morphological evolution or bradytely. However, for treeshrews it is not the reason they have been considered as “living fossils”. Instead, that designation was applied to treeshrews because they have been widely regarded as approximating the ancestral primate morphotype, despite the fact that various studies have concluded a very early divergence date between treeshrews, primates and other archontans^1^,^2^,^3^,^4^,^5^. As a superordinal group, Archonta traditionally includes primates, treeshrews, flying lemurs, plesiadapiforms, and bats^6^,^7^. Asher and Helgen (2010) modified the definition of Archonta by excluding bats from this superordinal group^1^, but some researchers prefer to use Euarchonta (originally based on molecular phylogeny without considering the monophyly of Archonta^8^) as the superordinal name for the group with bats excluded (e.g. refs 4, 7 and 9). Historically, treeshrews were classified as primates, but are now generally accepted as being among the closest living relatives of primates^2^,^4^,^10^,^11^,^12^.

Scandentia, the mammalian clade comprising treeshrews, is divided into the families Tupaiidae and Ptilocercidae^2^. Extant tupaiid treeshrews are moderately diverse (22 described species placed in two genera)^2^,^13^,^14^,^15^,^16^. In contrast, their sister group, Ptilocercidae, contains only one species, *Ptilocercus lowii*, which is the most plesiomorphic living treeshrew, and widely considered to be a “living model” of the ancestral primate^5^,^17^,^18^,^19^,^20^,^21^,^22^.

Although treeshrews have long been called “living fossils”^5^,^11^,^18^,^23^, fossils belonging to members of this group are extremely rare and mainly comprise small numbers of isolated teeth. Apart from the questionable late Middle Eocene “*Eodendrogale parva*”^24^, the earliest undoubted treeshrew fossils are no older than 18 Ma^24^,^25^,^26^, and only five fossil species have been named: *Tupaia sivalicus*, *T. miocenica*, *T. storchi*, *Prodendrogale yunnanica* and *P. engesseri*. All of these species are Miocene tupaiids, closely resembling their modern relatives^24^,^27^,^28^,^29^. The only previously known ptilocercid fossil is a late Miocene unnamed lower jaw fragment retaining m1 and the distal half of p4^30^. Here we report a small number of fossils referable to *Ptilocercus*, and closely resembling *P. lowii*, from the early Oligocene of China. With an age of approximately 34 million years, the fossil nearly doubles the temporal length of the known fossil record of treeshrews, and represents the oldest definitive fossil of a crown-group treeshrew.

These new treeshrew fossils were discovered at the Lijiawa mammalian fossil locality near Qujing City in Yunnan Province, China. Among the numerous fossil mammal specimens recovered from this fossil site are those belonging to a large form of *Gigantamynodon giganteus*, an unnamed species of *Cricetops*, and a primitive *Eucricetodon* comparable with *Eucricetodon caducus* from the earliest Oligocene of Xinjiang, China. Those species all indicate an early Oligocene age for this fauna^31^,^32^.

## Result


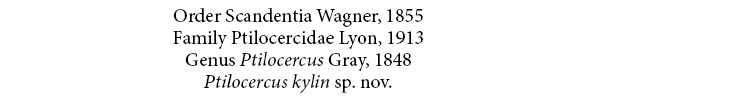


### Etymology

Specific epithet is derived from the name of Qilin District, in Qujing City. Qilin is the pinyin for kylin, a hoofed dragon-like beast of Chinese myth.

### Holotype

**Figure 1:**
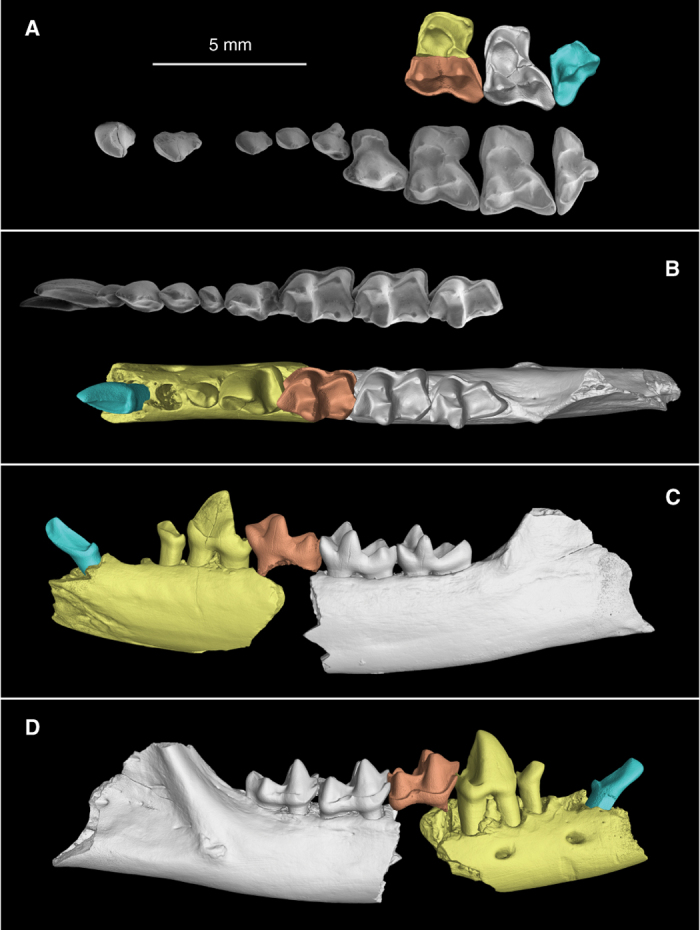
Upper and lower dentition (in color) of *Ptilocercus kylin* sp. nov., compared with *P. lowii* (USNM 32409, in gray-scale). (**A**) Crown view of the upper dentition. Fossils include the lingual half of a left M1 (IVPP V20689, reversed), the buccal half of a right M1 (IVPP V20690), a complete right M2 (IVPP V20691), and the buccal half of a left M3 (IVPP V20692, reversed). I1-2, C, P2-4, and M1-3 are shown for *P. lowii*. (**B**) Crown view of the lower dentition. Fossils include an isolated right lower canine (IVPP V20693), a right jaw fragment preserving p3-4 and the alveoli for i2-3, c, and p2 (IVPP V20694), an isolated right m1 (IVPP V20695), and a right jaw fragment preserving m2-3 (IVPP V20696, holotype). The alignment of the fossils is based on a left lower jaw fragment retaining a small portion of the i2-3 alveoli, alveoli and roots of c and p2, and p3-m3 (IVPP V20699, Fig. 2 and Supplementary Information). The i1-3, c, p2-4, and m1-3 are shown for *P. lowii*. (**C**), Lingual view of the lower dentition of *P. kylin*. (**D**), Buccal view of the dentition of *P. kylin*. Scale bar equals 5 mm.

IVPP V20696 (Fig. 1), a right mandibular fragment preserving m2 and m3.

### Hypodigm

IVPP V20689, lingual half of a left M1; IVPP V20690, buccal half of a right M1; IVPP V20691, a right M2; IVPP V20692, buccal half of a left M3; IVPP V20693, a right lower canine; IVPP V20694, a right jaw fragment preserving p3-4 and the alveoli for i2-3, c, and p2; IVPP V20695, a right m1; IVPP V 20697.1, buccal half of a left M1; IVPP V 20697.2, lingual half of a left M1; IVPP V 20697.3, lingual half of a left M2; IVPP V 20697.4, lingual half of a right M2; IVPP V 20697.5, a left c; IVPP V 20697.6, right p4; IVPP V 20697.7, a left m1; IVPP V 20697.8, a left m2; IVPP V 20697.9, a left m3; IVPP V 20698, a right jaw fragment preserving m2; IVPP V20699, a left lower jaw fragment retaining a small portion of the i2-3 alveoli, alveoli and roots of c and p2, and p3-m3.

### Locality and horizon

Lijiawa Mammalian Fossil locality, Yunnan Province, China. Earliest Oligocene, ~ 34 Ma.

### Diagnosis

Small treeshrew almost identical in size with *Ptilocercus lowii*. Differs from all tupaiids, but resembles *P. lowii* in: lacking dilambdodont upper molars; having a large hypocone on M1-2; strong cingulids on the lower molars; a p3 that is smaller than p2; and a premolariform lower canine. Differs from *P. lowii* in having: a relatively stronger paraconule and metaconule coupled with stronger pre- and post-conule cristae; a stronger lingual cingulum on M1-2; a relatively broader and taller p4 with less-developed paraconid, metaconid and talonid; and lacking a groove separating the hypoconulid and entoconid on the lower molars.

### Comparative morphology

The upper molars of ptilocercids are very different from those of tupaiid treeshrews^33^. All tupaiids have a V-shaped, buccally inclined paracone and metacone on each upper molar. The pre- and post-paracrista and the pre- and post-metacrista are buccally directed, forming the so-called dilambdodont tooth pattern. A mesostyle is always present, and is in contact with the postparacrista and premetacrista. The protocone of tupaiid treeshrews also is V-shaped and buccally inclined. The trigon basin enclosed by the preprotocrista and postprotocrista is very deep and narrow. The mesial, lingual and distal borders of the protocone are usually smooth, and no cingulum is present. By contrast, the molar cusps of *Ptilocercus kylin* and *Ptilocercus lowii* are rounded and basally expanded (Fig. 1). The preparacrista, postparacrista and premetacrista extend mesiodistally. The preprotocrista and postprotocrista of the protocone form a U-shape, surrounding a broad, shallow trigon basin. The combination of these features produces a typical bunodont tooth, a feature also traditionally considered to be characteristic for basal primates^33^,^34^,^35^.

The upper molars of *Ptilocercus kylin* are slightly different from those of *Ptilocercus lowii*. The lingual cingula on M1-2 of *P. kylin* are stronger, and the paraconules and metaconules have better developed pre- and post-cristae. The hypocones of M1-2 of *P. kylin* are proportionally smaller than in *P. lowii*, and as a result, the posterior borders of M1-2 are less indented in *P. kylin*. The buccal shelves are as broad in *P. kylin* as in *P. lowii*, but the buccal cingula are less developed in *P. kylin*. In *P. lowii*, the buccal cingula are very thick. They enclose two small folds between the preparacrista and postmetacrista. In *P. kylin*, no such folds are present. The metacone of M3 in *P*. *lowii* is conical with a round and expanded base, whereas in *P. kylin*, the cusp has a sharper appearance.

The lower dentition of ptilocercids also shows many characteristics that are not seen in tupaiid treeshrews. In tupaiids, the lower canine is a large peg-like tooth with a sharp apex. Their premolars are not closely spaced. The size of the premolars gradually increases from p2 to p4, and p3 has two roots. Their molars have trenchant cusps connected to one another by sharp crests. The molars usually have smooth buccal faces, and their trigonids are much higher than their talonids. By contrast, the lower canine of *Ptilocercus lowii* is a premolar-like tooth. It has a procumbent crown with a well-developed lingual cingulid and distal cusp, and has an obvious neck between the crown and root. The premolar region is shortened, and p2 and p3 are all single-rooted and oblique. The p3 is smaller than p2 and is much smaller than p4, a peculiar condition rarely seen in mammals. The lower molars of *P. lowii* are widely considered primate-like^33^,^34^,^35^. They have conical cusps connected by relatively weak crests. The buccal cingula of the lower molars, however, are very strong, and the trigonids of the molars are only slightly higher than the talonids. These characteristics that distinguish *P. lowii* from tupaiid treeshrews are all also present in *Ptilocercus kylin* (Fig. 1).

**Figure 2:**
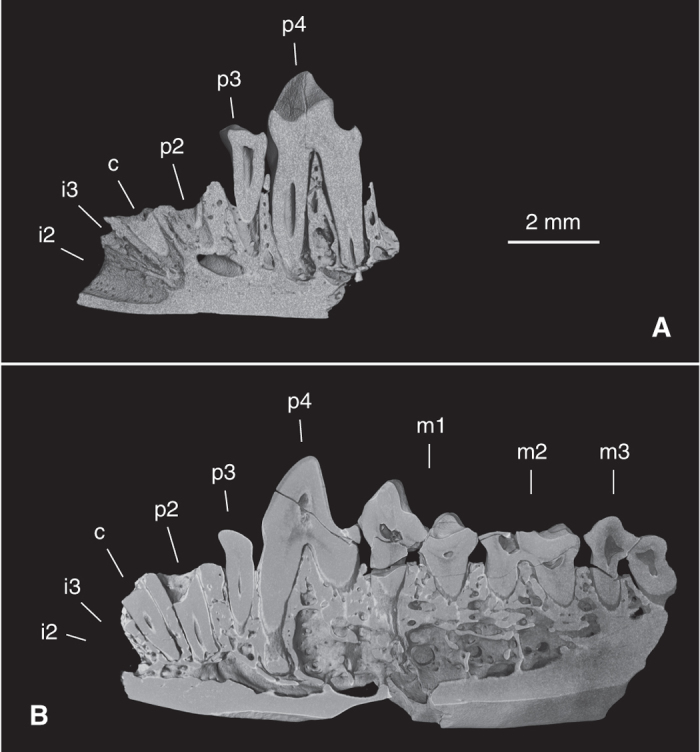
Sagittal sections through two lower jaw fragments of *Ptilocercus kylin* sp. nov. showing the tooth and alveolar loci. (**A**) a right lower jaw fragment retaining the alveoli of i2-3, c, alveoli and roots of c and p2, and p3-4 (IVPP V 20694); (**B**) a left lower jaw fragment retaining a small portion of the i2-3 alveoli, alveoli and roots of c and p2, and p3-m3 (IVPP V20699, reversed). Scale bar equals 2 mm.

One lower jaw fragment (IVPP V20694) preserves the alveoli of i2-3, the lower canine and p2 (Fig. 2). The alveolus of i2 is much larger than the i3 alveolus situated immediately distal to it. The orientation of both alveoli suggests that i2 and i3 were very procumbent. The canine alveolus is larger than those of i3 and p2, and the alveolus of p2 is in turn larger than that of p3 (Fig. 2). An isolated lower canine shows that this tooth is premolar-like, with a procumbent crown, an obvious neck between the crown and root, a strong lingual cingulid and a distal accessary cusp. Based on the relative sizes of the alveoli, p3 is much smaller than p4, and smaller than p2, a peculiar feature shared with *Ptilocercus lowii*. The p4 of *Ptilocercus kylin* is quite large, retaining a generalized morphology (with a very low paraconid and metaconid and a heel-like talonid) that is shared with tupaiids, insectivores and many stem primates. The paraconid and metaconid of this tooth are quite small, and are much lower than the sharp, trenchant protoconid. The talonid is not fully developed, forming only a short heel. However in *P. lowii*, p4 is relatively small, and is molariform, with a larger paraconid, a larger metaconid and a longer talonid. The combination of these features suggests that the p4 of *P. lowii* is specialized and represents an apomorphic condition. The lower molars of *P. kylin* are almost identical in shape and size with those of *P. lowii*, except for slight differences present at the distal ends of the teeth. Extant tupaiid and ptilocercid treeshrews all have a lingually positioned hypoconulid, which is separated from the entoconid by a sulcus. In *P. lowii*, the hypoconulid is more like a rounded terminal swelling of the lingually extended hypocristid, and the distal end of the entoconid is also rounded. Meanwhile in *P. kylin*, the hypoconulid is a cuspid, and is connected to the entoconid via short cristids. The m3 hypoconulid of *P. kylin* is relatively long and forms a short heel, and that feature is seen in many stem primates but not in *P. lowii* or other crown treeshrews.

## Discussion

**Figure 3:**
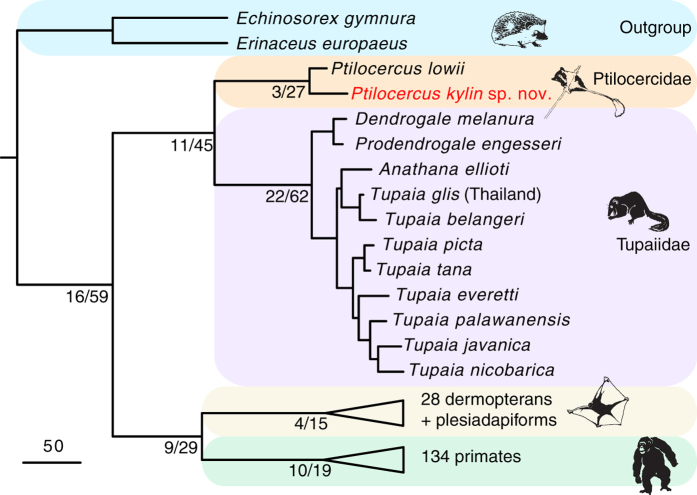
Summary phylogeny of treeshrews. Parsimony analysis is based on a data matrix including 1199 morphological characters and 658 molecular characters of long and short interspersed nuclear elements scored for 130 fossil and 47 living taxa. The topology of extant treeshrews, flying lemurs and primates used as a backbone constraint or “molecular scaffold” is based on a gene supermatrix (ref. 50). Numbers before the slashes at the internodes are the absolute Bremer Support values; numbers after the slashes are Relative Bremer Support values. The strict consensus of the unconstrained analysis is provided in the Supplementary Information. The topologies of treeshrews in both the constrained and unconstrained analysis are identical. Scale bar equals 50 character changes.

Although a few diagnostic traits distinguish *Ptilocercus kylin* from *Ptilocercus lowii*, the two species are exceptionally similar to each other. Therefore, it is unsurprising that a phylogenetic analysis based on large data matrix placed *P. kylin* as the sister species to *P. lowii* (Fig. 3). The monophyly of the *Ptilocercus* branch is strongly supported (Supplementary Information). At the same time, the monophyly of the tupaiid clade also has significant character support. Within Tupaiidae, the interspecific relationships are similar to those based on phylogenetic analysis of molecular data^5^,^36^,^37^,^38^. More specifically, our analysis also supports referring “*Urogale*” *everetti* to the genus *Tupaia*^5^.

Various methods of phylogenetic inference and molecular divergence date estimation have suggested that the split between treeshrews and their close relatives (primates and colugos), and that between Ptilocercidae and Tupaiidae (within treeshrews) occurred in the Palaeogene^3^,^4^,^5^. The inter-generic diversification within tupaiid treeshrews began as early as 35 Ma^5^. Detailed morphological comparisons between the early Oligocene *Ptilocercus kylin* and extant *Ptilocercus lowii* reveal very few diagnostic differences that could support placing these species in separate genera. *Ptilocercus* treeshrews appear to have evolved at a very slow pace (bradytely) and accumulated very few morphological changes over the last 34 million years. In many ways they are even more conservative than their sister group Tupaiidae. Given its very ancient divergence time from its sister group, the known species diversity within Ptilocercidae is roughly ten times lower than that in Tupaiidae. Only three ptilocercid taxa (*P. lowii*, *P. kylin*, and a late Miocene unnamed fossil from Yunnan China) are known at present, in comparison to approximately thirty known living and fossil tupaiid taxa. However, the species diversity of Tupaiidae itself is quite low when compared to primates.

**Figure 4:**
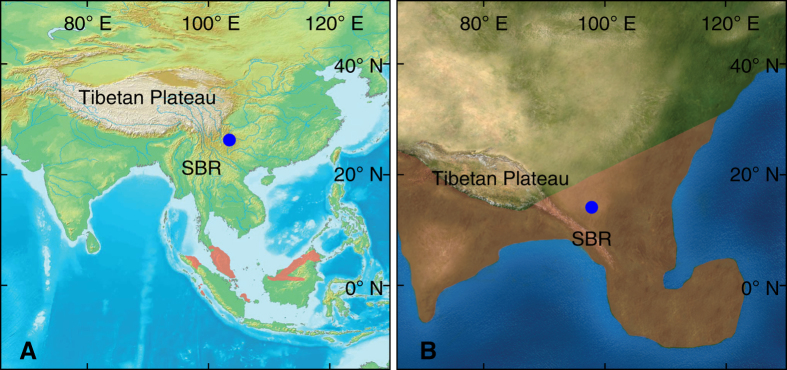
*Ptilocercus* treeshrew distribution in the context of southern Asia’s modern geography and early Oligocene palaeogeography. (**A**) Fossil locality of *Ptilocercus kylin* sp. nov. (blue dot) and the distribution of the living species *Ptilocercus lowii* (pale reddish shading). The background map is from: https://upload.wikimedia.org/wikipedia/commons/c/cf/WorldMap-A_non-Frame.png (under the Creative Commons Share Alike license: https://creativecommons.org/licenses/by-sa/3.0/deed.en). (**B**) Fossil locality (blue dot) and reconstructed palaeogeographic distribution of the closed canopy of tropical rain forest and monsoonal forest (pale reddish shading) in the early Oligocene. The palaeogeographic reconstruction is from ref. 46 (Nature Publishing Group License: 3646200322068). The position of the fossil locality on the palaeogeographic reconstruction was estimated based on its distance from the Tibetan Plateau and the Sino-Burman Ranges (SBR). The distribution of the tropical rain forest and monsoonal forest is from ref. 47.

The extant *Ptilocercus lowii* is arboreal and nocturnal, and occurs largely in tropical rainforest areas with abundant vines and undergrowth in southern Thailand, Malaysia, Brunei, and Indonesia (Fig. 4)^39^. Individuals of that species consume large amounts of alcoholic nectar, and have a symbiotic pollination relationship with the tropical bertam palm (thought to be at least 55 million years old)^40^. The conserved morphology of *Ptilocercus* clade, as revealed by the specimens of *Ptilocercus kylin*, could be the result of a relatively stable ecological niche in the forests of Southeastern Asia through the Cenozoic^40^,^41^,^42^, and conversely, the morphological similarity between the fossil and extant *Ptilocercus* also may suggest that *P. kylin* occupied a dense tropical rainforest habitat, like its living sister species.

The Eocene-Oligocene transition was marked by dramatic changes in global climate and sea level^43^,^44^,^45^, and those changes are associated with the expansion of open habitats, and a major retraction of tropical rainforest to low latitudes. However in south Asia, mid-latitude areas were connected to the equatorial region by uninterrupted terrestrial habitats^46^, and closed canopy tropical rainforests and monsoonal forests still extended over a vast area during this transitional period^47^. The presence of early Oligocene *Ptilocercus* far to the north of its current range is consistent with the hypothesis that rainforest environments were much more widespread in Asia in the early Oligocene than they are today (Fig. 4). We posit that the geographic distribution of *Ptilocercus* treeshrews has tracked the expansion and retraction of tropical forests in southern Asia through the Cenozoic.

## Methods

### Measurements

Specimens were measured under the ZEN Pro 2012 system stored with a Zeiss stero-microscope (Discovery V20), and were calibrated from the caliper. The results are listed in Supplementary Information S-Table 1.

### Phylogenetic methods

The phylogenetic analysis was based on a data matrix derived from published data in ref. 12. In total, 1857 characters were scored for 177 taxa. The 1857 characters comprise 485 dental, 202 cranial, 309 postcranial, 203 soft tissue characters, and 658 molecular characters. Two erinaceid insectivores, *Erinaceus europaeus* and *Echinosorex gymnura*, were selected as outgroup taxa. The ingroup comprises 13 treeshrews, 28 flying lemurs and plesiadapiforms, 45 lemuriforms and adapiforms, 40 tarsiiforms, and 49 anthropoids. Of these taxa, 47 are extant species. The character-taxon matrix is available on the online database MorphoBank (Project 2152). The data matrix was edited in Mesquite v3.03 software^48^ and saved in the NEXUS format. Specimens checked and scored and the arguments for the characters are listed as notes in the NEXUS file.

Parsimony analysis of the total evidence dataset (dental, cranial, postcranial, soft tissue, and molecular data) was undertaken using TNT, Tree analysis using New Technology, a parsimony analysis program subsidized by the Willi Hennig Society^49^. We ran multiple replications, using sectorial searches, drifting, ratchet and fusing combined (Supplementary Information: S-Table 2). Random sectorial search, constraint sectorial search and exclusive sectorial search were used. Ten cycles of tree drifting, 10 cycles of ratchet and 10 cycles of tree fusing were performed in the search. Default parameter settings for random sectorial search, constraint sectorial search, exclusive sectorial search, tree drifting, ratchet and fusing were used. The search level was set as 10 for 177 taxa. Optimal scores were searched with 10000 replications. Some characters are set as ordered (listed in the Supplementary Information), but the outgroups were not used as reference for ordering the character states. All characters have equal weight. The relationships of some extant taxa (based on Springer *et al.*’s gene super-matrix)^50^ were used as a backbone constraint or molecular “scaffold”, but the relationships among the treeshrews were not constrained.

Approximately 20 hours were required to finish the unconstrained search. More than 769 billion rearrangements were examined. Forty-seven trees with a best score of 13067 were retained. Nearly 21 hours were required to finish the backbone-constrained search. More than 728 billion rearrangements were examined. Twenty trees with a best score of 13141 were retained. The best trees and the strict consensus of these trees were described in PAUP*^51^. The results of backbone-constrained and unconstrained searches were compared in S-Table 3. The backbone-constrained trees (Supplementary Information: S-Fig. 3) are preferred because they incorporated more phylogenetically-relevant information (morphological and molecular information).

We used absolute Bremer support and Relative Bremer Support^52^,^53^, calculated in TNT (Supplementary Information: S-Table 4), to describe the stability of the phylogenetic result (Supplementary Information: S-Fig. 3, S-Fig. 4).

We define the Order Scandentia as a crown group, including the clade stemming from the most common ancestor of extant ptilocercids and tupaiids. We described the strict consensus tree of the 20 most parsimonious trees from the backbone-constrained searches in PAUP*^51^. The ACCTRAN (accelerated transformation^51^,^54^) method of character-state optimization is used. Gaps are treated as “missing”. Multistate taxa interpretation depends on “{}” versus “()” designation (“min” values for CI, RI, and RC are minimum-possible character lengths). Synapomorphies for Ptilocercidae, Tupaiidae and Scandentia are listed in the supplementary tables (Supplementary Information: S-Table 5 to S-Table 7). The character numbers listed in S-Table 5 to S-Table 7 correspond to those in the NEXUS file.

## Additional Information

**How to cite this article**: Li, Q. and Ni, X. An early Oligocene fossil demonstrates treeshrews are slowly evolving "living fossils". *Sci. Rep.* **6**, 18627; doi: 10.1038/srep18627 (2016).

## Supplementary Material

Supplementary Information

Supplementary Dataset 1

Supplementary Dataset 2

## References

[b1] Asher R. & Helgen K. Nomenclature and placental mammal phylogeny. BMC Evolutionary Biology 10, 102, doi: 10.1186/1471-2148-10-102 (2010). 20406454 PMC2865478

[b2] Helgen K. M. in Mammal Species of the World. A Taxonomic and Geographic Reference, Third edition (eds Wilson D. E. & Reeder D. M.) 104–109 (The Johns Hopkins University Press, 2005).

[b3] Janecka J. E. *et al.* Molecular and genomic data identify the closest living relative of primates. Science 318, 792–794 (2007). 17975064 10.1126/science.1147555

[b4] O’Leary M. A. *et al.* The placental mammal ancestor and the post–K-Pg radiation of placentals. Science 339, 662–667 (2013). 23393258 10.1126/science.1229237

[b5] Roberts T. E., Lanier H. C., Sargis E. J. & Olson L. E. Molecular phylogeny of treeshrews (Mammalia: Scandentia) and the timescale of diversification in Southeast Asia. Molecular Phylogenetics and Evolution 60, 358–372 (2011). 21565274 10.1016/j.ympev.2011.04.021

[b6] McKenna M. C. & Bell S. K. Classification of Mammals, above the Species Level. (Columbia University Press, 1997).

[b7] Rose K. D. The Beginning of the Age of Mammals. 1–428 (The Johns Hopkins University Press, 2006).

[b8] Waddell P. J., Cao Y., Hauf J. & Hasegawa M. Using novel phylogenetic methods to evaluate mammalian mtDNA, including amino acid-invariant sites-logdet plus site stripping, to detect internal conficts in the data, with special reference to the positions of hedgehog, armadillo, and elephant. Syst. Biol. 48, 31–53 (1999). 12078643 10.1080/106351599260427

[b9] Silcox M. T., Bloch J. I., Sargis E. J. & Boyer D. M. in The Rise of Placental Mammals (eds Rose K. D. & Archibald J. D.) 127–144 (The Johns Hopkins University Press, 2005).

[b10] Bloch J. I., Silcox M. T., Boyer D. M. & Sargis E. J. New Paleocene skeletons and the relationship of plesiadapiforms to crown-clade primates. Proc. Natl. Acad. Sci. USA 104, 1159–1164 (2007). 17229835 10.1073/pnas.0610579104 PMC1783133

[b11] Luckett W. P. Comparative Biology and Evolutionary Relationships of Tree Shrews. 1–314 (Plenum Press, 1980).

[b12] Ni X. *et al.* The oldest known primate skeleton and early haplorhine evolution. Nature 498, 60–64 (2013). 23739424 10.1038/nature12200

[b13] Sargis E. J., Woodman N., Reese A. T. & Olson L. E. Using hand proportions to test taxonomic boundaries within the *Tupaia glis* species complex (Scandentia, Tupaiidae). J. Mammal. 94, 183–201 (2013).

[b14] Sargis E. J., Woodman N., Morningstar N. C., Reese A. T. & Olson L. E. Morphological distinctiveness of Javan *Tupaia hypochrysa* (Scandentia, Tupaiidae). J. Mammal. 94, 938–947 (2013).

[b15] Sargis E. J., Woodman N., Morningstar N. C., Reese A. T. & Olson L. E. Island history affects faunal composition: the treeshrews (Mammalia: Scandentia: Tupaiidae) from the Mentawai and Batu Islands, Indonesia. Biological Journal of the Linnean Society 111, 290–304 (2014).

[b16] Sargis E. J., Campbell K. K. & Olson L. E. Taxonomic boundaries and craniometric variation in the treeshrews (Scandentia, Tupaiidae) from the Palawan faunal region. J. Mammal. Evol. 21, 111–123 (2014).

[b17] Sargis E. J. A preliminary qualitative analysis of the axial skeleton of tupaiids (Mammalia, Scandentia): functional morphology and phylogenetic implications. J. Zool., Lond. 253, 473–483 (2001).

[b18] Sargis E. J. The postcranial morphology of *Ptilocercus lowii* (Scandentia, Tupaiidae): an analysis of primatomorphan and volitantian characters J. Mammal. Evol. 9, 137–160 (2002).

[b19] Sargis E. J. Functional morphology of the hindlimb of tupaiids (Mammalia, Scandentia) and its phylogenetic implications J. Morphol. 254, 149–185 (2002). 12353299 10.1002/jmor.10025

[b20] Sargis E. J. Functional morphology of the forelimb of Tupaiids (Mammalia, Scandentia) and its phylogenetic implications. J. Morphol. 253, 10–42 (2002). 11981802 10.1002/jmor.1110

[b21] Sargis E. J. in Primate Origins: Adaptations and Evolution (eds Ravosa M. J. & Dagosto M.) 51–82 (Springer, 2007).

[b22] Szalay F. S. & Drawhorn G. in Comparative Biology and Evolutionary Relationships of Tree Shrews (ed Luckett W. P) 133–169 (Plenum Press, 1980).

[b23] Tattersall I. in Living Fossils (eds Eldredge N. & Stanley S. M.) 32–37 (Springer, Verlag, 1984).

[b24] Ni X. & Qiu Z. Tupaiine tree shrews (Scandentia, Mammalia) from the Yuanmou *Lufengpithecus* locality of Yunnan, China. Swiss Journal of Palaeontology 131, 51–60 (2012).

[b25] Mein P. & Ginsburg L. Les mammifères du gisement miocène inférieur de Li Mae Long, Thaïlande: systématique, biostratigraphie et paléoenvironnement. Geodiversitas 19, 783–844 (1997).

[b26] Qiu Z. Fossil tupaiid from the hominoid locality of Lufeng, Yunnan. Vertebrata PalAsiatica 24, 308–319 (1986).

[b27] Jacobs L. L. in Comparative Biology and Evolutionary Relationships of Tree Shrews (ed Luckett W. P.) 205–216 (Plenum Press, 1980).

[b28] Luckett W. P. & Jacobs L. L. Proposed fossil tree shrew genus *Palaeotupaia*. Nature 288, 104 (1980).

[b29] Sargis E. J. New views on tree shrews: the role of tupaiids in primate supraordinal relationships. Evol. Anthropol. 13, 56–66 (2004).

[b30] Ni X. & Qiu Z. The micromammalian fauna from the Leilao, Yuanmou hominoid locality: Implications for biochronology and paleoecology. J. Hum. Evol. 42, 535–546 (2002). 11969296 10.1006/jhev.2001.0540

[b31] Maridet O. & Ni X. A new cricetid rodent from the early Oligocene of Yunnan, China, and its evolutionary implications for early Eurasian cricetids. J. Vert. Paleontol. 33, 185–194 (2013).

[b32] Ni X., Meng J., Wu W. & Ye J. A new Early Oligocene peradectine marsupial (Mammalia) from the Burqin region of Xinjiang, China. Naturwissenschaften 94, 237–241 (2007). 17136514 10.1007/s00114-006-0182-2

[b33] Butler P. M. in Comparative Biology and Evolutionary Relationships of Tree Shrews (ed Patrick Luckett W.) 171–204 (Plenum Press, 1980).

[b34] Le Gros Clark W. E. On the anatomy of the pen-tailed tree-shrew (*Ptilocercus lowii*). Proceedings of the Zoological Society of London 96, 1179–1309 (1926).

[b35] Le Gros Clark W. E. The Antecedents of Man, an Introduction of the Evolution of the Primates. 1–374 (Edinburgh University Press, 1959).

[b36] Olson L. E., Sargis E. J. & Martin R. D. Phylogenetic relationships among treeshrews (scandentia): a review and critique of the morphological evidence. J. Mammal. Evol. 11, 49–71 (2004).

[b37] Olson L. E., Sargis E. J. & Martin R. A. Intraordinal phylogenetics of treeshrews (Mammalia: Scandentia) based on evidence from the mitochondrial 12S rRNA gene. Molecular Phylogenetics and Evolution 35, 656–673 (2005). 15878134 10.1016/j.ympev.2005.01.005

[b38] Roberts T. E., Sargis E. J. & Olson L. E. Networks, Trees, and treeshrews: assessing support and identifying conflict with multiple loci and a problematic root. Systematic Biology 58, 257–270 (2009). 20525582 10.1093/sysbio/syp025 PMC2715937

[b39] Han K. H. & Stuebing R. *Ptilocercus lowii*. *The IUCN Red List of Threatened Species* **2008**, e.T41491A10467786, http://dx.doi.org/10.2305/IUCN.UK.2008.RLTS.T41491A10467786.en (2008) (Date of access: 30/06/2008).

[b40] Wiens F. *et al.* Chronic intake of fermented floral nectar by wild treeshrews. Proc. Natl. Acad. Sci. USA 105, 10426–10431 (2008). 18663222 10.1073/pnas.0801628105 PMC2492458

[b41] Sussman R. W. & Raven P. H. Pollination by lemurs and marsupials: Archaic coevolutionary system. Science 200, 731–736 (1978). 17743224 10.1126/science.200.4343.731

[b42] Sussman R. W., Tab Rasmussen D. & Raven P. H. Rethinking primate origins again. Am. J. Primatol. 75, 95–106 (2013). 23184701 10.1002/ajp.22096

[b43] Jaramillo C., Rueda M. J. & Mora G. Cenozoic plant diversity in the neotropics. Science 311, 1893–1896 (2006). 16574860 10.1126/science.1121380

[b44] Morley R. J. Origin and Evolution of Tropical Rain Forests. 1–362 (Wiley, 2000).

[b45] Zachos J., Pagani M., Sloan L., Thomas E. & Billups K. Trends, rhythms, and aberrations in global climate 65 ma to present. Science 292, 686–693 (2001). 11326091 10.1126/science.1059412

[b46] Licht A. *et al.* Asian monsoons in a late Eocene greenhouse world. Nature 513, 501–506 (2014). 25219854 10.1038/nature13704

[b47] Morley R. J. in Tropical Rainforest Responses to Climatic Change, Second edition (eds Bush M., Flenley J. & Gosling W.) 1–34 (Springer-Verlag, 2011).

[b48] Maddison W. P. & Maddison D. R. *Mesquite: A modular system for evolutionary analysis*. Version 3.03, http://mesquiteproject.org (2015).

[b49] Goloboff P. A., Farris J. S. & Nixon K. C. TNT, a free program for phylogenetic analysis. Cladistics 24, 774–786 (2008).

[b50] Springer M. S. *et al.* Macroevolutionary dynamics and historical biogeography of primate diversification inferred from a species supermatrix. PLoS ONE 7, e49521, doi: 10.1371/journal.pone.0049521 (2012). 23166696 PMC3500307

[b51] Swofford D. L. *PAUP*. Phylogenetic Analysis Using Parsimony (*and Other Methods)*. Version 4.0 Beta, http://www.paup.csit.fsu.edu (Sinauer Associates, Sunderland, 2002).

[b52] Bremer K. Branch support and tree stability. Cladistics 10, 295–304 (1994).

[b53] Goloboff P. A. & Farris J. S. Methods for quick consensus estimation. Cladistics 17, S26–S34 (2001).

[b54] Swofford D. L. & Maddison W. P. Reconstructing ancestral character states under Wagner parsimony. Mathematical Biosciences 87, 199–229 (1987).

