# Supplementary material for: An early Oligocene fossil demonstrates treeshrews are slowly evolving “living fossils”
Source: Sci Rep. 2016 Jan 14;6:18627. doi: 10.1038/srep18627 (PMC4725336; doi:10.1038/srep18627)
Supplement: Supplementary Information [file srep18627-s1.pdf]

Supplementary Information for

An early Oligocene fossil demonstrates treeshrews are slowly evolving "living fossils"

Qiang Li[1,2], Xijun Ni[1,2]

1. Key Laboratory of Vertebrate Evolution and Human Origins, Institute of Vertebrate Paleontology and Paleoanthropology, Chinese Academy of Sciences, 142 Xi Zhi Men Wai Street, Beijing, 100044, China
2. CAS Center for Excellence in Tibetan Plateau Earth Sciences, Beijing, 100101, China
Correspondence to: nixijun@ivpp.ac.cn

This PDF file includes:

Figures S1 to S5
Tables S1 to S7

Other Supplementary Materials for this paper includes the following:

Supplementary Datasets as TNT and NEXUS files:
Ni_2015_Treeshrews_TNT.doc
Ni_2015_Treeshrews_NEXUS.doc

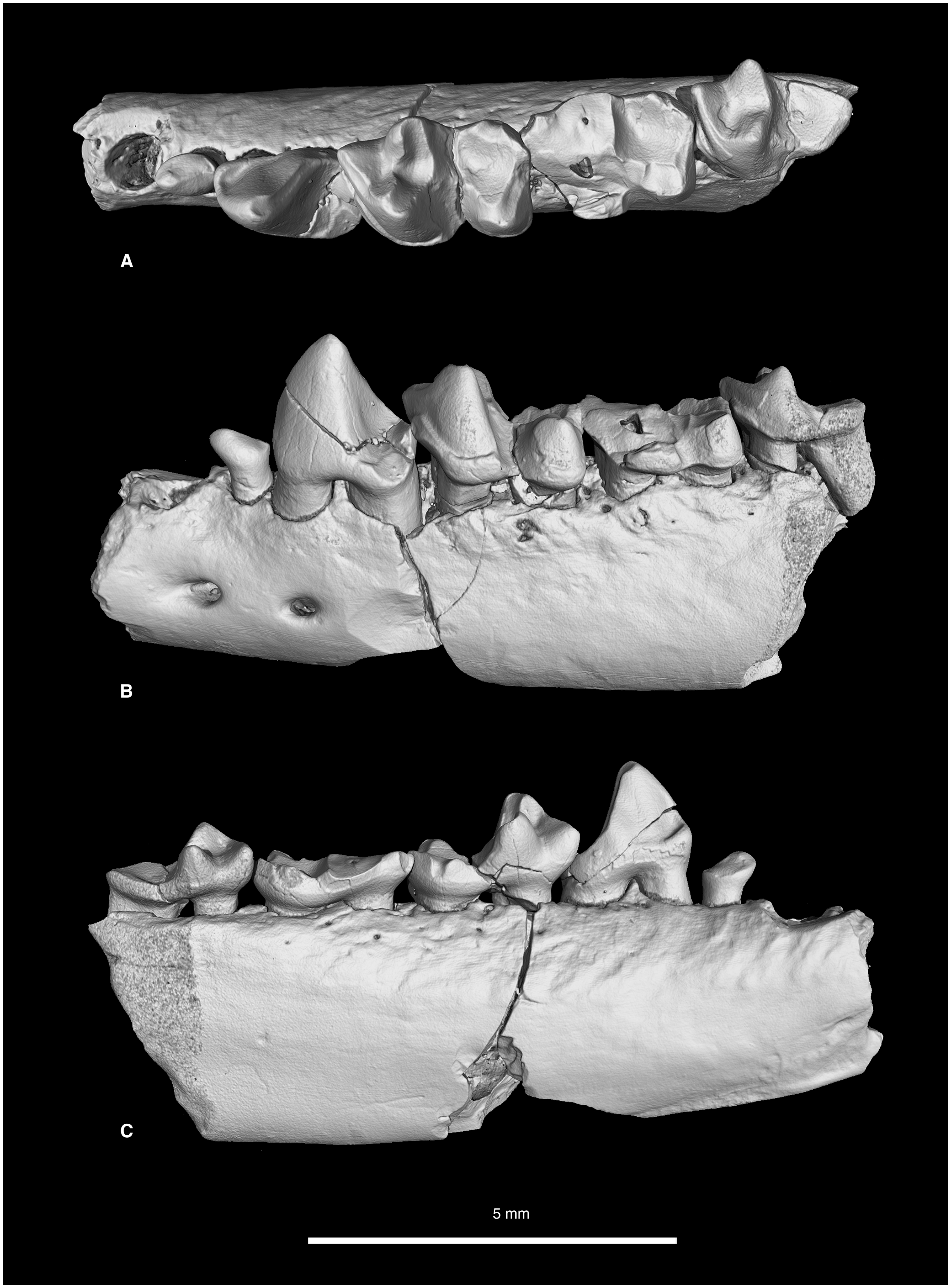
A
B
C
5 mm

Figure S1.

Crown (**A**), buccal (**B**) and lingual (**C**) views of a left jaw fragment of *Ptilocercus kylin* sp. nov., retaining a small portion of the i2-3 alveoli, alveoli and roots of c and p2, and p3-m3 (IVPP V20699).

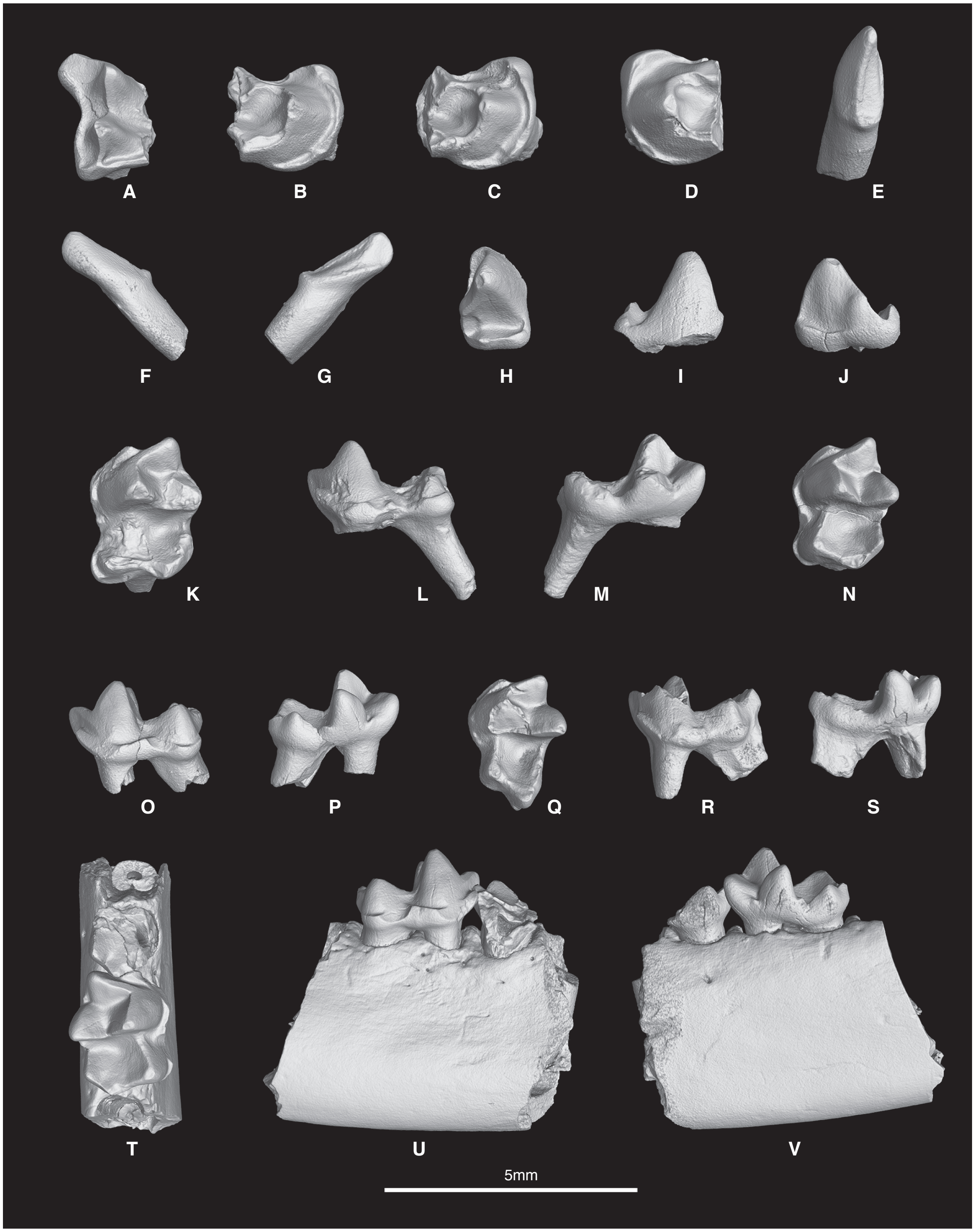
A
B
C
D
E
F
G
H
I
J
K
L
M
N
O
P
Q
R
S
T
U
V
5mm

Figure S2

Additional specimens of *Ptilocercus kylin* sp. nov. **A**, buccal half of a left M1, IVPP V 20697.1, crown view; **B**, lingual half of a left M1, IVPP V 20697.2, crown view; **C**, lingual half of a left M2, IVPP V 20697.3, crown view; **D**, lingual half of a right M2, IVPP V 20697.4, crown view; **E-G**, left c, IVPP V 20697.5, crown, buccal and lingual views; **H-J**, right p4, IVPP V 20697.6, crown, buccal and lingual views; **K-M**, left m1, IVPP V 20697.7, crown, buccal and lingual views; **N-P**, left m2, IVPP V 20697.8, crown, buccal and lingual views; **Q-S**, left m3, IVPP V 20697.9, crown, buccal and lingual views; **T-V**, right jaw fragment keeping m2, IVPP V 20698, crown, buccal and lingual views. Scale bar indicates 5 mm.

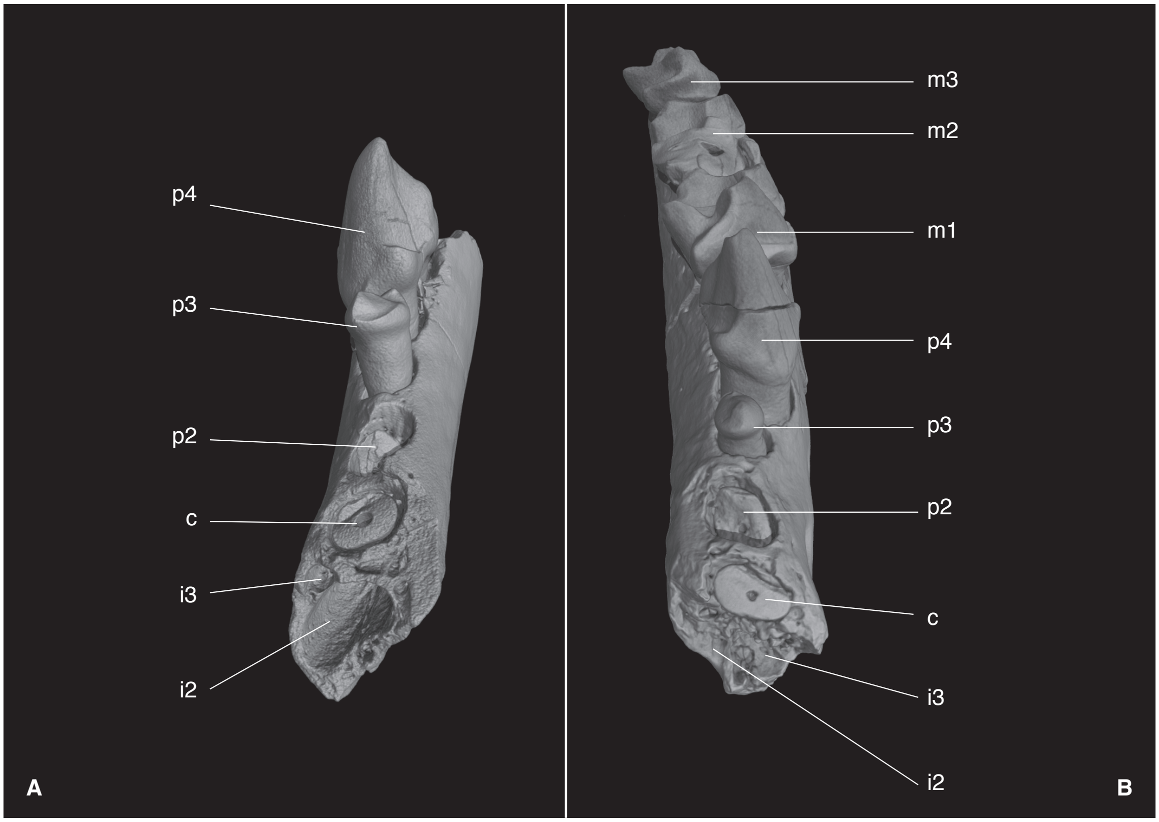

Figure S3

Rostral-crown view of two lower jaw fragments of *Ptilocercus kylin* sp. nov. showing the tooth and alveolar loci. **A**, a right lower jaw fragment retaining the alveoli of i2-3, c, alveoli and root s of c and p2, and p3-4 (IVPP V 20694); **B**, a left lower jaw fragment retaining a small portion of the i2-3 alveoli, alveoli and roots of c and p2, and p3-m3 (IVPP V20699).

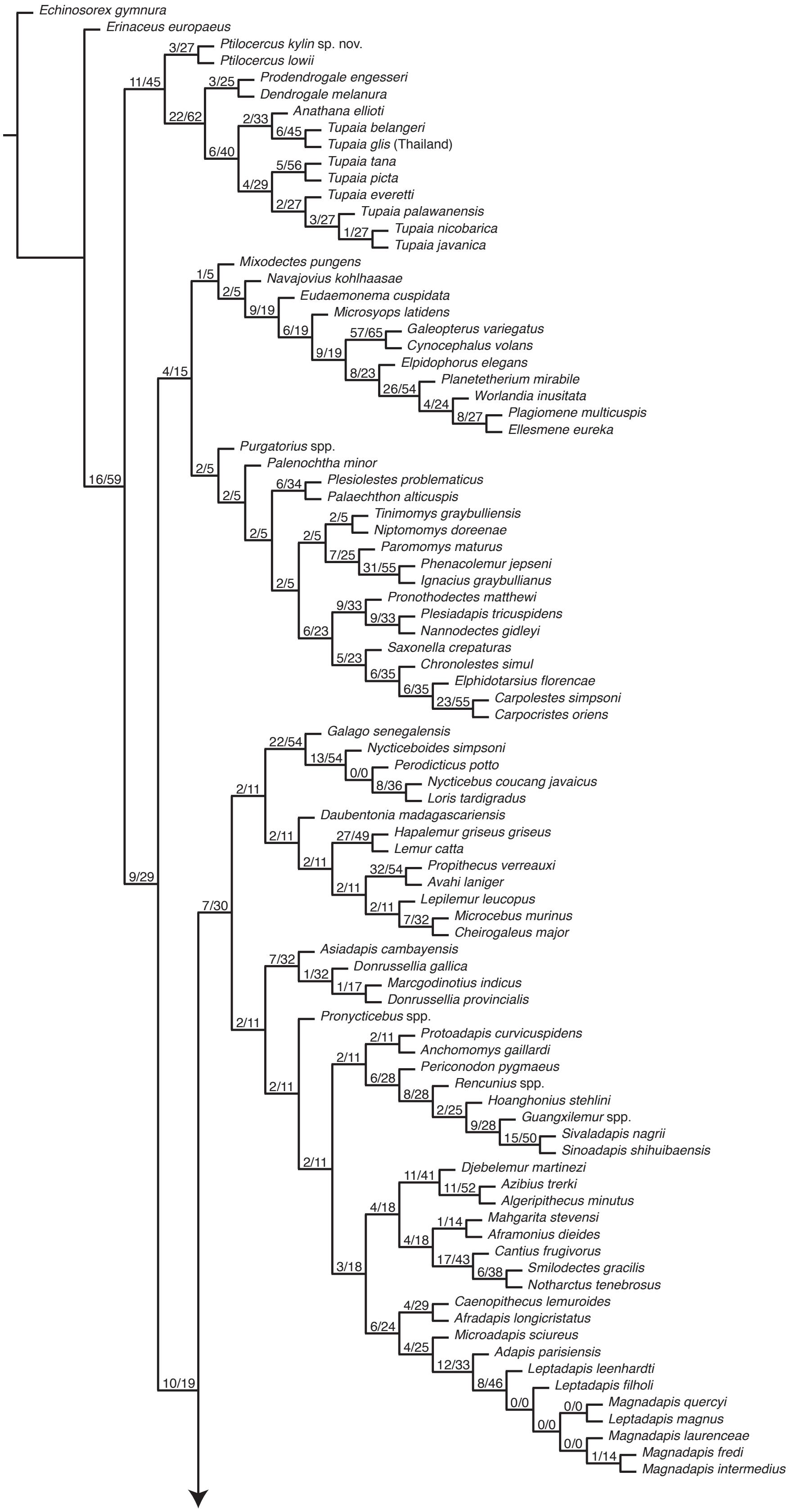
Echinosorex gymnura
Erinaceus europaeus
3/27
Ptilocercus kylin sp. nov.
Ptilocercus lowii
11/45
3/25
Prodendrogale engesseri
Dendrogale melanura
22/62
2/33
Anathana ellioti
6/45
Tupaia belangeri
Tupaia glis (Thailand)
6/40
5/56
Tupaia tana
Tupaia picta
4/29
Tupaia everetti
2/27
3/27
Tupaia palawanensis
1/27
Tupaia nicobarica
Tupaia javanica
Mixodectes pungens
1/5
Navajovius kohlhaasae
2/5
Eudaemonema cuspidata
9/19
Microsyops latidens
6/19
57/65
Galeopterus variegatus
9/19
Cynocephalus volans
Elpidophorus elegans
8/23
Planetetherium mirabile
26/54
Worlandia inusitata
4/15
4/24
Plagiomene multicuspis
8/27
Ellesmene eureka
Purgatorius spp.
Palenochtha minor
2/5
6/34
Plesiolestes problematicus
Palaechthon alticuspis
2/5
2/5
Tinimomys graybulliensis
2/5
Niptomomys doreenae
2/5
7/25
Paromomys maturus
16/59
31/55
Phenacolemur jepseni
Ignacius graybullianus
2/5
9/33
Pronothodectes matthewi
9/33
Plesiadapis tricuspidens
6/23
Nannodectes gidleyi
Saxonella crepaturas
5/23
Chronolestes simul
6/35
Elphidotarsius florencae
6/35
23/55
Carpolestes simpsoni
Carpocristes oriens
Galago senegalensis
22/54
Nycticeboides simpsoni
13/54
0/0
Perodicticus potto
8/36
Nycticebus coucang javaicus
Loris tardigradus
2/11
Daubentonia madagascariensis
2/11
27/49
Hapalemur griseus griseus
Lemur catta
2/11
32/54
Propithecus verreauxi
9/29
2/11
Avahi laniger
Lepilemur leucopus
7/30
2/11
7/32
Microcebus murinus
Cheirogaleus major
Asiadapis cambayensis
7/32
Donrussellia gallica
1/32
1/17
Marcgodinotius indicus
Donrussellia provincialis
2/11
Pronycticebus spp.
2/11
Protoadapis curvicuspidens
Anchomomys gaillardi
2/11
Periconodon pygmaeus
6/28
Rencunius spp.
8/28
2/11
Hoanghonius stehlini
2/25
Guangxilemur spp.
9/28
Sivaladapis nagrii
15/50
Sinoadapis shihuibaensis
2/11
Djebelemur martinezi
11/41
Azibius trerki
11/52
Algeripithecus minutus
4/18
Mahgarita stevensi
1/14
Aframonius dieides
4/18
Cantius frugivorus
17/43
Smilodectes gracilis
6/38
3/18
Notharctus tenebrosus
Caenopithecus lemuroides
4/29
Afradapis longicristatus
6/24
Microadapis sciureus
4/25
Adapis parisiensis
12/33
Leptadapis leenhardti
8/46
Leptadapis filholi
10/19
0/0
0/0
Magnadapis quercyi
0/0
Leptadapis magnus
Magnadapis laurenceae
0/0
1/14
Magnadapis fredi
Magnadapis intermedius

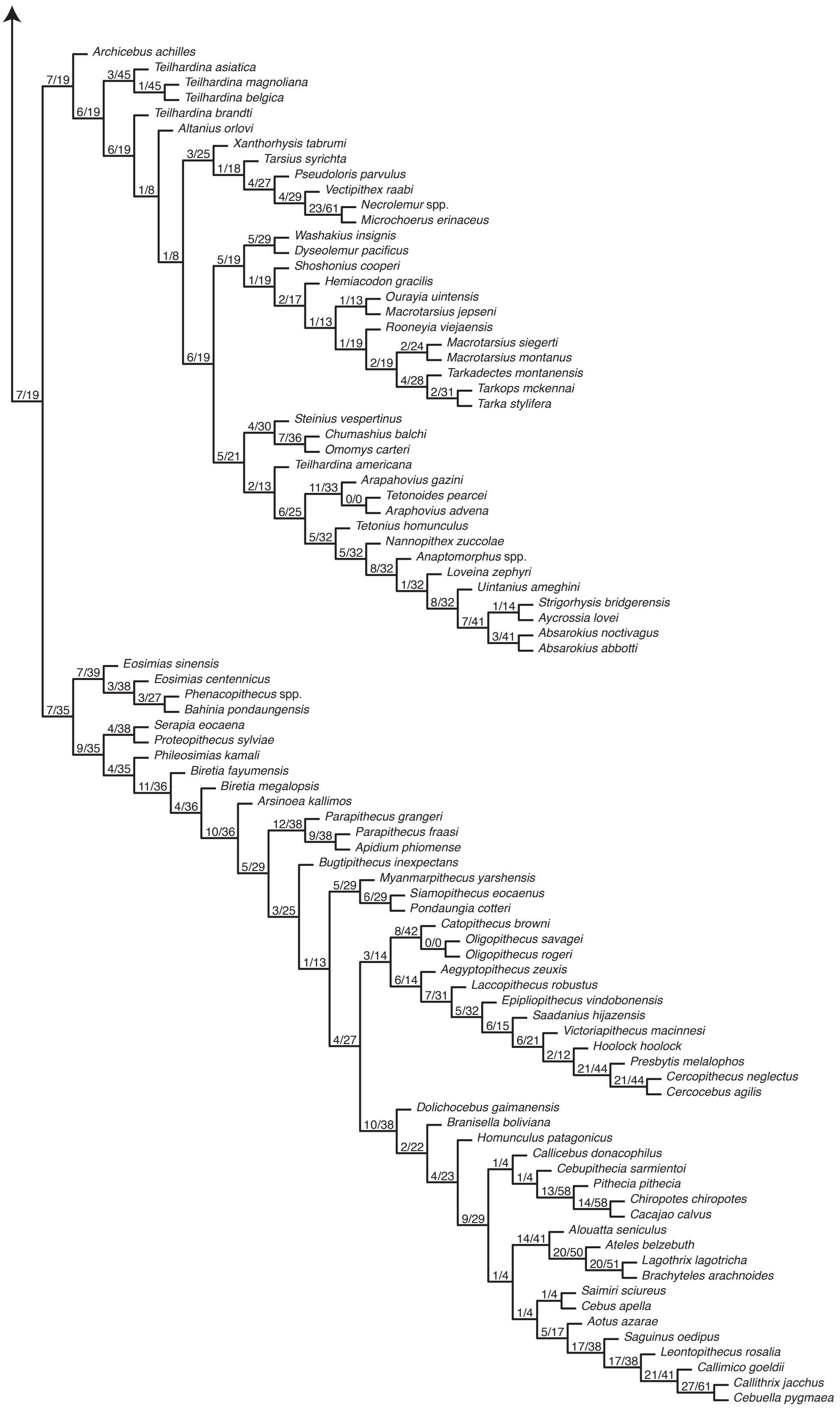
Archicebus achilles
Teilhardina asiatica
Teilhardina magnoliana
Teilhardina belgica
Teilhardina brandti
Altanius orlovi
Xanthorhysis tabrumi
Tarsius syrichta
Pseudoloris parvulus
Vectipithex raabi
Necrolemur spp.
Microchoerus erinaceus
Washakius insignis
Dyseolemur pacificus
Shoshonius cooperi
Hemiacodon gracilis
Ourayia uintensis
Macrotarsius jepseni
Rooneyia viejaensis
Macrotarsius siegerti
Macrotarsius montanus
Tarkadectes montanensis
Tarkops mckennai
Tarka stylifera
Steinius vespertinus
Chumashius balchi
Omomys carteri
Teilhardina americana
Arapahovius gazini
Tetonoides pearcei
Araphovius advena
Tetonius homunculus
Nannopithex zuccolae
Anaptomorphus spp.
Loveina zephyri
Uintanius ameghini
Strigorhysis bridgerensis
Aycrossia lovei
Absarokius noctivagus
Absarokius abbotti
Eosimias sinensis
Eosimias centennicus
Phenacopithecus spp.
Bahinia pondaungensis
Serapia eocaena
Proteopithecus sylviae
Phileosimias kamali
Biretia fayumensis
Biretia megalopsis
Arsinoea kallimos
Parapithecus grangeri
Parapithecus fraasi
Apidium phiomense
Bugtipithecus inexpectans
Myanmarpithecus yarshensis
Siamopithecus eocaenus
Pondaungia cotteri
Catopithecus browni
Oligopithecus savagei
Oligopithecus rogeri
Aegyptopithecus zeuxis
Laccopithecus robustus
Epipliopithecus vindobonensis
Saadanius hijazensis
Victoriapithecus macinnesi
Hoolock hoolock
Presbytis melalophos
Cercopithecus neglectus
Cercocebus agilis
Dolichocebus gaimanensis
Branisella boliviana
Homunculus patagonicus
Callicebus donacophilus
Cebupithecia sarmientoi
Pithecia pithecia
Chiropotes chiropotes
Cacajao calvus
Alouatta seniculus
Ateles belzebuth
Lagothrix lagotricha
Brachyteles arachnoides
Saimiri sciureus
Cebus apella
Aotus azarae
Saguinus oedipus
Leontopithecus rosalia
Callimico goeldii
Callithrix jacchus
Cebuella pygmaea
7/19
7/19
6/19
3/45
1/45
6/19
1/8
1/8
3/25
1/18
4/27
4/29
23/61
5/29
5/19
1/19
2/17
1/13
1/13
1/19
2/19
2/24
4/28
2/31
6/19
4/30
7/36
5/21
2/13
11/33
0/0
6/25
5/32
5/32
8/32
1/32
8/32
7/41
1/14
3/41
7/39
3/38
3/27
7/35
4/38
9/35
4/35
11/36
4/36
10/36
12/38
9/38
5/29
3/25
5/29
6/29
1/13
8/42
0/0
3/14
6/14
7/31
5/32
6/15
6/21
2/12
21/44
21/44
4/27
10/38
2/22
4/23
1/4
1/4
13/58
14/58
9/29
14/41
20/50
20/51
1/4
1/4
1/4
5/17
17/38
17/38
21/41
27/61

Figure S4

Bremer supports for the strict consensus tree from backbone-constrained phylogenetic analysis. Numbers before the slashes are the Bremer Support values; numbers after the slashes are Relative Supports.

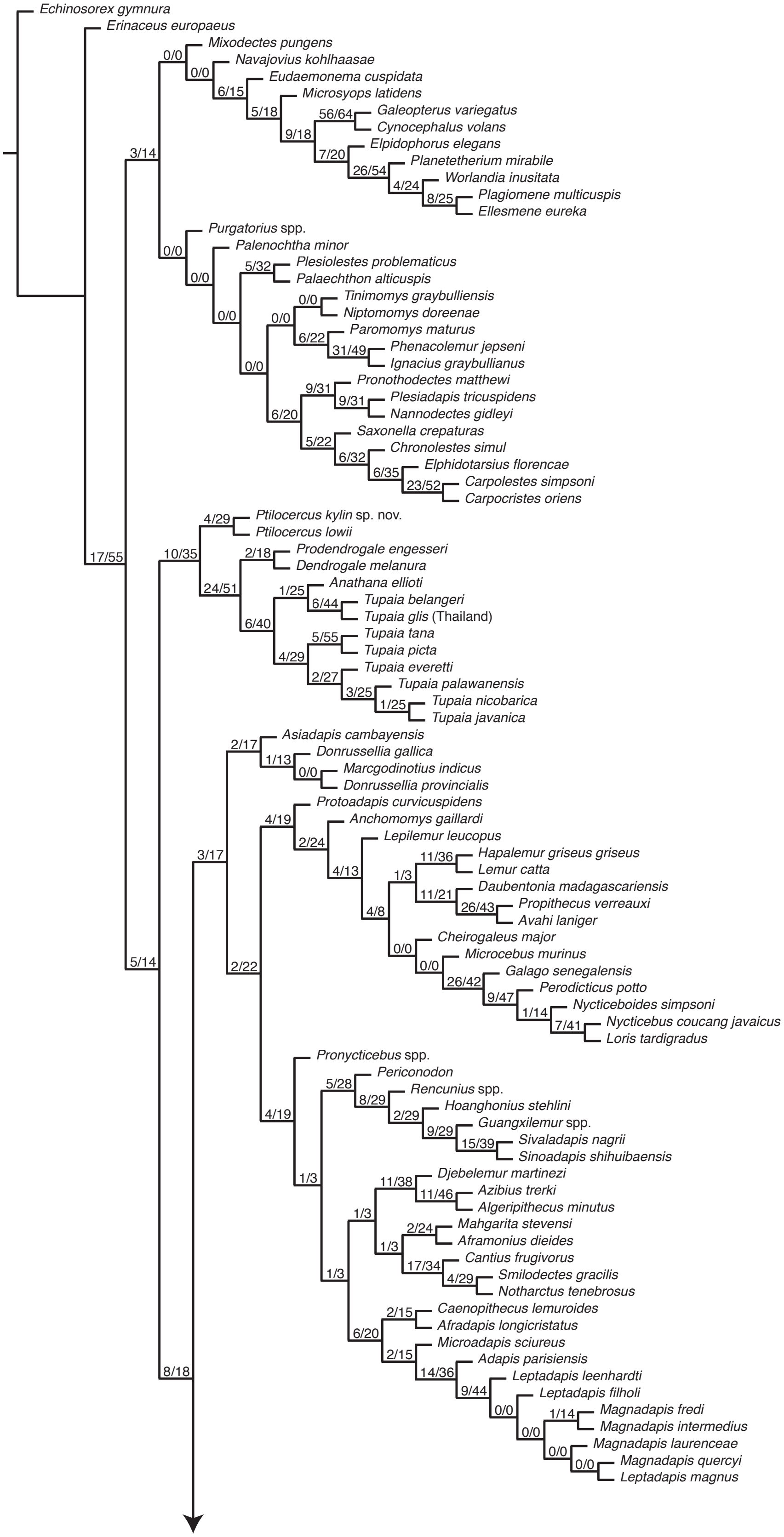
Echinosorex gymnura
Erinaceus europaeus
Mixodectes pungens
Navajovius kohlhaasae
Eudaemonema cuspidata
Microsyops latidens
Galeopterus variegatus
Cynocephalus volans
Elpidophorus elegans
Planetetherium mirabile
Worlandia inusitata
Plagiomene multicuspis
Ellesmene eureka
Purgatorius spp.
Palenochtha minor
Plesiolestes problematicus
Palaechthon alticuspis
Tinimomys graybulliensis
Niptomomys doreenae
Paromomys maturus
Phenacolemur jepseni
Ignacius graybullianus
Pronothodectes matthewi
Plesiadapis tricuspidens
Nannodectes gidleyi
Saxonella crepaturas
Chronolestes simul
Elphidotarsius florencae
Carpolestes simpsoni
Carpocristes oriens
Ptilocercus kylin sp. nov.
Ptilocercus lowii
Prodendrogale engesseri
Dendrogale melanura
Anathana ellioti
Tupaia belangeri
Tupaia glis (Thailand)
Tupaia tana
Tupaia picta
Tupaia everetti
Tupaia palawanensis
Tupaia nicobarica
Tupaia javanica
Asiadapis cambayensis
Donrussellia gallica
Marcgodinotius indicus
Donrussellia provincialis
Protoadapis curvicuspidens
Anchomomys gaillardi
Lepilemur leucopus
Hapalemur griseus griseus
Lemur catta
Daubentonia madagascariensis
Propithecus verreauxi
Avahi laniger
Cheirogaleus major
Microcebus murinus
Galago senegalensis
Perodicticus potto
Nycticeboides simpsoni
Nycticebus coucang javaicus
Loris tardigradus
Pronycticebus spp.
Periconodon
Rencunius spp.
Hoanghonius stehlini
Guangxilemur spp.
Sivaladapis nagrii
Sinoadapis shihuibaensis
Djebelemur martinezi
Azibius trerki
Algeripithecus minutus
Mahgarita stevensi
Aframonius dieides
Cantius frugivorus
Smilodectes gracilis
Notharctus tenebrosus
Caenopithecus lemuroides
Afradapis longicristatus
Microadapis sciureus
Adapis parisiensis
Leptadapis leenhardti
Leptadapis filholi
Magnadapis fredi
Magnadapis intermedius
Magnadapis laurenceae
Magnadapis quercyi
Leptadapis magnus
0/0
0/0
6/15
5/18
56/64
9/18
7/20
26/54
4/24
8/25
3/14
0/0
0/0
5/32
0/0
0/0
0/0
6/22
31/49
0/0
9/31
9/31
6/20
5/22
6/32
6/35
23/52
4/29
10/35
2/18
24/51
1/25
6/44
6/40
5/55
4/29
2/27
3/25
1/25
17/55
2/17
1/13
0/0
4/19
2/24
11/36
4/13
1/3
11/21
26/43
4/8
3/17
0/0
0/0
26/42
9/47
1/14
7/41
5/14
2/22
5/28
8/29
2/29
9/29
15/39
4/19
11/38
11/46
1/3
1/3
2/24
1/3
17/34
4/29
1/3
2/15
6/20
2/15
14/36
9/44
0/0
1/14
0/0
0/0
0/0
8/18

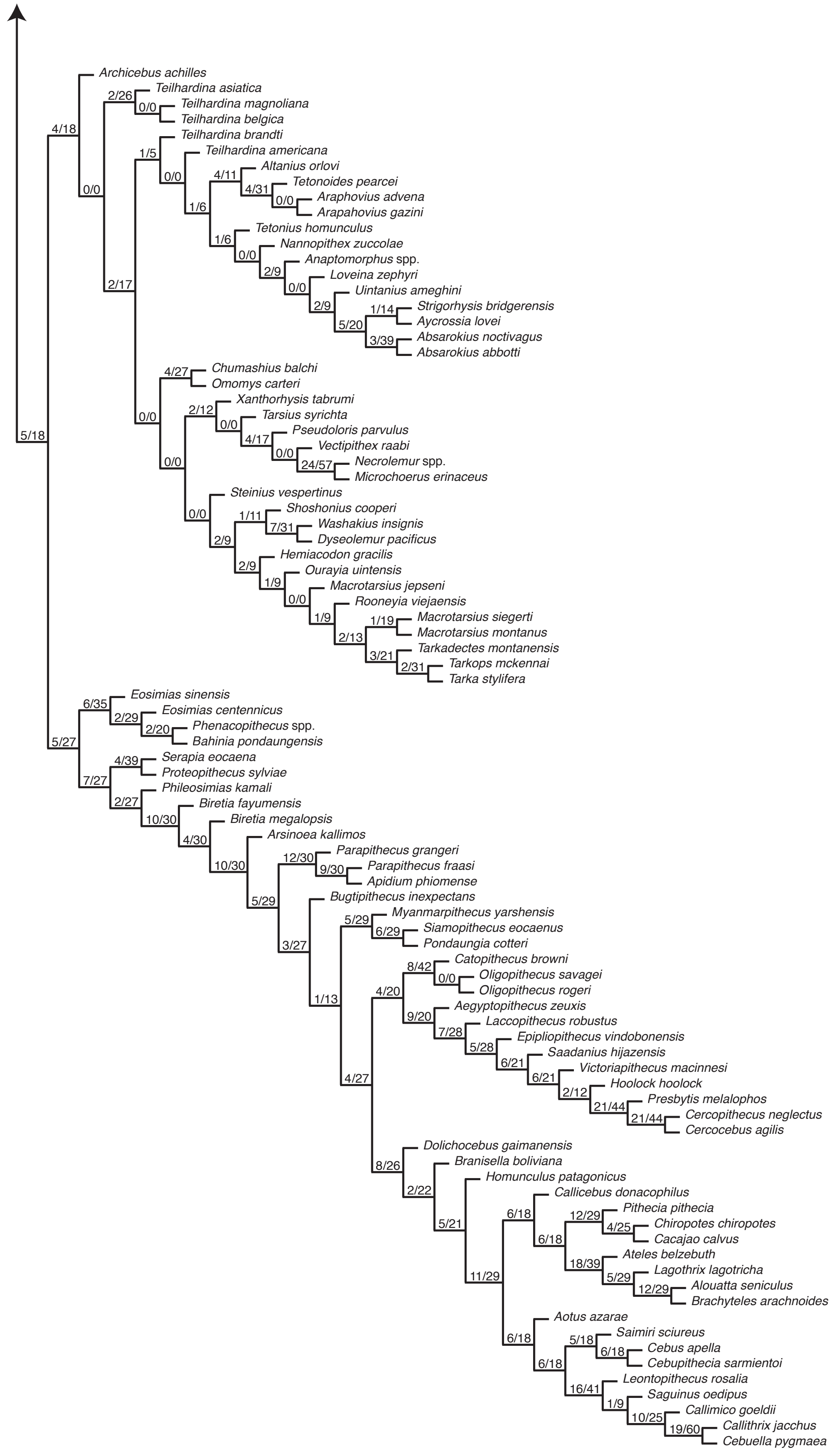
Archicebus achilles
Teilhardina asiatica
Teilhardina magnoliana
Teilhardina belgica
Teilhardina brandti
Teilhardina americana
Altanius orlovi
Tetonoides pearcei
Araphovius advena
Arapahovius gazini
Tetonius homunculus
Nannopithex zuccolae
Anaptomorphus spp.
Loveina zephyri
Uintanius ameghini
Strigorhysis bridgerensis
Aycrossia lovei
Absarokius noctivagus
Absarokius abbotti
Chumashius balchi
Omomys carteri
Xanthorhysis tabrumi
Tarsius syrichta
Pseudoloris parvulus
Vectipithex raabi
Necrolemur spp.
Microchoerus erinaceus
Steinius vespertinus
Shoshonius cooperi
Washakius insignis
Dyseolemur pacificus
Hemiacodon gracilis
Ourayia uintensis
Macrotarsius jepseni
Rooneyia viejaensis
Macrotarsius siegerti
Macrotarsius montanus
Tarkadectes montanensis
Tarkops mckennai
Tarka stylifera
Eosimias sinensis
Eosimias centennicus
Phenacopithecus spp.
Bahinia pondaungensis
Serapia eocaena
Proteopithecus sylviae
Phileosimias kamali
Biretia fayumensis
Biretia megalopsis
Arsinoea kallimos
Parapithecus grangeri
Parapithecus fraasi
Apidium phiomense
Bugtipithecus inexpectans
Myanmarpithecus yarshensis
Siamopithecus eocaenus
Pondaungia cotteri
Catopithecus browni
Oligopithecus savagei
Oligopithecus rogeri
Aegyptopithecus zeuxis
Laccopithecus robustus
Epipliopithecus vindobonensis
Saadanius hijazensis
Victoriapithecus macinnesi
Hoolock hoolock
Presbytis melalophos
Cercopithecus neglectus
Cercocebus agilis
Dolichocebus gaimanensis
Branisella boliviana
Homunculus patagonicus
Callicebus donacophilus
Pithecia pithecia
Chiropotes chiropotes
Cacajao calvus
Ateles belzebuth
Lagothrix lagotricha
Alouatta seniculus
Brachyteles arachnoides
Aotus azarae
Saimiri sciureus
Cebus apella
Cebupithecia sarmientoi
Leontopithecus rosalia
Saguinus oedipus
Callimico goeldii
Callithrix jacchus
Cebuella pygmaea
5/18
4/18
0/0
2/26
0/0
1/5
0/0
4/11
4/31
0/0
1/6
1/6
0/0
2/9
0/0
2/9
5/20
1/14
3/39
2/17
4/27
0/0
2/12
0/0
4/17
0/0
24/57
0/0
0/0
1/11
7/31
2/9
2/9
1/9
0/0
1/9
2/13
1/19
3/21
2/31
5/27
6/35
2/29
2/20
7/27
4/39
2/27
10/30
4/30
10/30
5/29
12/30
9/30
3/27
1/13
5/29
6/29
4/27
4/20
8/42
0/0
9/20
7/28
5/28
6/21
6/21
2/12
21/44
21/44
8/26
2/22
5/21
11/29
6/18
6/18
12/29
4/25
18/39
5/29
12/29
6/18
6/18
5/18
6/18
16/41
1/9
10/25
19/60

Figure S5

Bremer supports for the strict consensus tree from non-constraint phylogenetic analysis. Numbers before the slashes are the Bremer Support values; numbers after the slashes are Relative Supports.

Table S1.

Measurements of the specimens

Specimen No.	Specimen	Length (mm)	Width (mm)
IVPP V20697.1	Left upper M1 buccal half	2.34	-
IVPP V20697.2	Left upper M1 lingual half	-	-
IVPP V20697.3	Left upper M2 lingual half	-	-
IVPP V20697.4	Right upper M2 lingual half	-	-
IVPP V20697.5	Left lower canine	1.81	0.93
IVPP V20697.6	Right lower p4	1.94	1.42
IVPP V20697.7	Left lower m1	2.76	2.00
IVPP V20697.8	Left lower m2	2.42	1.74
IVPP V20697.9	Left lower m3	2.47	1.70
IVPP V20698	Right lower fragmental mandible with m2: m2	2.49	1.70
IVPP V20699	Left lower fragmental mandible with p3-m3: p3	0.92	0.67
IVPP V20699	Left lower fragmental mandible with p3-m3: p4	2.08	1.45
IVPP V20699	Left lower fragmental mandible with p3-m3: m1	2.70	2.01
IVPP V20699	Left lower fragmental mandible with p3-m3: m2	2.49	1.83
IVPP V20699	Left lower fragmental mandible with p3-m3: m3	2.20	1.71
IVPP V20690	Right upper M1 buccal half	2.70	-
IVPP V20689	Left upper M1 lingual half	-	-
IVPP V20691	Right upper M2	2.36	3.30
IVPP V20692	Right upper M3 buccal half	2.11	-
IVPP V20693	Right lower canine	1.79	0.99
IVPP V20694	Right lower fragmental mandible with p3-p4: p3	1.05	0.86
IVPP V20694	Right lower fragmental mandible with p3-p4: p4	2.20	1.65
IVPP V20695	Right lower m1	2.59	1.77
IVPP V20696	Right lower fragmental mandible with m2-m3: m2	2.55	1.92
IVPP V20696	Right lower fragmental mandible with m2-m3: m3	2.61	1.81

Table S2.

TNT script for running multiple replications, using sectorial searches, drifting, ratchet and fusing combined

```
log Ni_2015_treeshrews.log;
mxram 1000;
p Ni_2015_treeshrews_TNT.txt;
hold 100000;
coll tbr;
report +/1;
tsave * Ni_2015_treeshrews.tre;
xm=level 10 177 rep 10000 fuse 10 dri 10 rss css xss hit 1 rat 10 xmix;
save;
tsave/;
log/;
zzz;
```

Table S3.

Comparison between the results from backbone-constrained searches and non-constraint searches

	Without constraints	Backbone constrained
Replications	10000	10000
Most parsimonious tree number	47	20
Searching time	19:59:50	20:53:09
Rearrangements examined	769,986,524,849	728,067,644,730
Tree length	13067	13141
Consensus Method	Strict	Strict
Tree length of consensus	14124	13914
Consistency Index of consensus	0.2110	0.2142
Retention Index of consensus	0.7182	0.7235
Ptilocercidae monophyly	supported	supported
Tupaiidae monophyly	supported	supported
Scandentia monophyly	supported	supported
Plesiadapiformes-Dermoptera monophyly	supported	supported
Primates monophyly	supported	supported
Primates-Scandentia monophyly	supported	not supported
Archonta monophyly	supported	supported

Table S4.

TNT script for calculating the Bremer Supports and Relative Bremer Supports

```
log Ni_2015_treeshrews_Bremer.log;
mxram 4000;
p Ni_2015_treeshrews_TNT.txt;
hold 100000;
sub 100 ;
ttag= ;
p Ni_2015_treeshrews.tre ;
bsupp !!+0 1. ;
sub 100x0.90 ;
bsupp [!!+0 1.;
ttag;
ttag & Ni_2015_treeshrews_Bremer.svg;
log/;
zzz;
```

Table S5.

Synapomorphic characters supporting the Ptilocercidae clade

Character	Steps	CI	Change
4 (I 4: Upper I1 root orient)	1	0.059	0 --> 1
13 (I 13: Upper I1 mesioling)	1	0.100	0 --> 1
28 (I 28: Upper I2 distal he)	1	0.125	1 --> 0
32 (I 31: Two-rooted upper i)	1	0.500	1 --> 0
45 (C 10: Distal heel on upp)	1	0.267	0 --> 1
57 (P 7: Upper P2 length rel)	1	0.091	0 --> 1
58 (P 8: Upper P2 length rel)	1	0.088	0 --> 1
63 (P 13: Upper P2 metastyle)	1	0.138	0 --> 1
65 (P 15: Upper P2 lingual c)	1	0.118	1 --> 0
68 (P 18: Upper P2 distal ci)	1	0.062	0 --> 1
76 (P 25: Upper P3 size rela)	1	0.118	1 --> 0
77 (P 26: Upper P3 paracone)	1	0.135	2 --> 0
80 (P 29: Upper P3 protocone)	1	0.059	0 --> 1
113 (P 60: Upper P3 "Nannopi)	1	0.120	1 --> 0
118 (P 65: Upper P4 buccal-l)	1	0.125	2 --> 1
129 (P 76: Upper P4 metastyl)	1	0.094	1 --> 2
140 (P 87: Upper P4 mesial c)	1	0.105	1 --> 0
168 (M 2: Upper M1 hypocone)	1	0.109	1 --> 2
212 (M 44: Upper M1-2 lingua)	1	0.190	0 --> 1
258 (i 2: Lower i1 size)	1	0.083	1 ==> 0
267 (i 11: Lower incisor occ)	1	0.111	0 --> 1
286 (c 2: Lower canine size)	1	0.056	1 ==> 2
287 (c 3: Lower canine heigh)	1	0.129	1 ==> 0
296 (c 12: Long axis of the)	1	0.143	0 ==> 1
304 (p 7: Lower p2 size rela)	2	0.136	0 ==> 2
305 (p 8: Lower p2 crown hei)	2	0.158	0 --> 2
316 (p 19: Lower p3 length r)	1	0.095	1 --> 0
322 (p 25: Lower p3 lingual)	1	0.074	0 ==> 1
339 (p 42: Lower p3 root num)	1	0.059	0 ==> 1
340 (p 43: Lower p3 orientat)	1	0.143	0 ==> 1
358 (p 61: Lower p4 postmeta)	1	0.034	0 ==> 1
360 (p 63: Lower p4 mesiolin)	1	0.059	0 --> 1
404 (m 18: Lower m1 position)	1	0.116	1 ==> 0
470 (m 83: Lower m1-2 distob)	1	0.136	1 --> 2
488 (Cranial 3: Anteroposter)	1	0.231	0 --> 1
506 (Cranial 21: Mastoid for)	1	0.500	0 --> 1
517 (Cranial 32: Epitympanic)	1	0.111	0 --> 1
519 (Cranial 34: Bony tube e)	1	0.333	0 --> 1
531 (Cranial 46: Medial expo)	1	1.000	0 --> 1
556 (Cranial 70: Suprameatal)	1	0.500	0 --> 1
560 (Cranial 74: Orbital con)	1	0.188	0 --> 1

561 (Cranial 75: Obital fron)	1	0.150	1 --> 0
686 (Mandibular 17: Orientat)	1	0.045	0 --> 1
696 (Vertebral 9: Number of)	1	0.200	0 --> 2
700 (Vertebral 13: Ventrodor)	1	0.250	1 --> 2
703 (Vertebral 16: Accessory)	1	0.200	1 --> 0
707 (Vertebral 20: Width of)	1	0.167	0 --> 1
713 (Costal 1: Rib craniocau)	1	0.333	0 --> 1
716 (Scapular 3: Coracoid pr)	1	0.250	0 --> 1
719 (Scapular 6: Spine tilt)	1	0.214	0 --> 1
740 (Humeral 6: Orientation)	1	0.111	1 --> 0
743 (Humeral 9: Anterior sur)	1	0.091	0 --> 1
744 (Humeral 10: Zona conoid)	1	0.211	0 --> 1
748 (Humeral 14: Anterior tr)	1	0.222	0 --> 1
749 (Humeral 15: Posterior u)	1	0.500	2 --> 1
751 (Humeral 17: Medial epic)	1	0.105	0 --> 1
763 (Humeral 29: Lesser tube)	1	0.167	1 --> 0
796 (Carpal 6: Centrale arti)	1	0.333	0 --> 1
800 (Carpal 10: Lunate artic)	1	0.200	0 --> 1
807 (Carpal 17: Spiral proxi)	1	0.500	0 --> 1
814 (Innominate 2: Divergenc)	1	0.111	0 --> 1
815 (Innominate 3: Width of)	1	0.250	2 --> 1
818 (Innominate 6: Anterior)	1	0.211	1 --> 2
822 (Innominate 10: Ischiac)	1	0.304	1 --> 0
823 (Innominate 11: Ischiac)	1	0.150	1 --> 0
830 (Innominate 18: Pubic le)	2	0.280	0 --> 2
854 (Femoral 20: Lateral con)	1	0.167	1 --> 0
855 (Femoral 21: Relative si)	1	0.133	0 --> 1
856 (Femoral 22: Medial bulg)	1	0.100	0 --> 1
865 (Tibial 4: Shape of dist)	1	0.333	0 --> 1
870 (Tibial 9: Shape of dist)	1	0.133	1 --> 2
883 (Astragalar 1: Height of)	1	0.087	1 --> 2
897 (Astragalar 14: Talar he)	1	0.077	1 --> 0
901 (Astragalar 18: Talar bo)	1	0.125	0 --> 1
906 (Calcaneal 4: Relative l)	1	0.211	1 --> 0
918 (Calcaneal 16: Plantar t)	1	0.125	0 --> 1
923 (Navicular 2: Position o)	1	0.333	0 --> 1
924 (Navicular 3: Expansion)	1	0.400	0 --> 1
934 (Cuneiform 2: Entocuneif)	1	0.143	0 --> 1
937 (Cuneiform 5: Medial ext)	1	0.222	0 --> 1

Table S6.

Synapomorphic characters supporting the Tupaiidae clade

Characters	Steps	CI	Change
33 (I 32: Diastema between u)	1	0.200	1 ==> 2
48 (C 13: Diastema between u)	1	0.167	2 ==> 1
85 (P 34: Upper P3 preparacr)	1	0.059	1 --> 0
86 (P 35: Upper P3 pronounce)	1	0.250	1 ==> 0
126 (P 73: Upper P4 preparac)	1	0.069	2 --> 0
173 (M 6: Upper M1 paraconul)	1	0.200	1 ==> 2
174 (M 7: Upper M1 preparaco)	2	0.061	0 --> 2
181 (M 14: Upper M2 hypocone)	1	0.088	1 --> 0
185 (M 18: Upper M2 metacone)	1	0.077	0 ==> 2
190 (M 22: Upper M2 paraconu)	1	0.205	1 ==> 2
191 (M 23: Upper M2 preparac)	2	0.057	0 --> 2
215 (M 47: Upper M1-2 parast)	1	0.169	1 ==> 2
220 (M 52: Upper M1-2 prepar)	1	0.240	0 ==> 2
221 (M 53: Upper M1-2 postpa)	1	0.115	2 ==> 0
224 (M 56: Upper M1-2 mesost)	3	0.323	0 ==> 3
225 (M 57: Upper M1-2 stylar)	1	0.120	2 ==> 3
235 (M 66: Upper M1-2 prepro)	1	0.048	0 ==> 1
239 (M 70: Upper M1-2 paraco)	1	0.091	0 --> 1
240 (M 71: Upper M1-2 mesial)	2	0.259	0 ==> 2
241 (M 72: Upper M1-2 distal)	2	0.364	0 ==> 2
246 (M 77: Upper M1-2 connec)	1	0.125	0 ==> 2
250 (M 81: Upper M1-2 deep s)	1	0.333	0 ==> 1
263 (i 7: Tooth comb)	1	0.333	0 ==> 1
292 (c 8: Diastema between c)	1	0.154	2 ==> 1
325 (p 28: Lower p3 paraconi)	1	0.214	0 --> 1
348 (p 51: Lower p4 metaconi)	1	0.136	1 ==> 2
350 (p 53: Lower p4 metaconi)	1	0.125	1 --> 0
351 (p 54: Lower p4 paraconi)	1	0.070	1 ==> 2
355 (p 58: Lower p4 buccal p)	1	0.074	2 --> 1
407 (m 21: Lower m1 buccal c)	2	0.075	2 ==> 0
408 (m 22: Lower m1 mesiobuc)	2	0.093	2 --> 0
409 (m 23: Lower m1 hypoflex)	1	0.043	1 ==> 2
426 (m 39: Lower m2 position)	1	0.278	0 ==> 1
429 (m 42: Lower m2 buccal c)	2	0.098	2 ==> 0
430 (m 43: Lower m2 mesiobuc)	2	0.095	2 --> 0
431 (m 44: Lower m2 hypoflex)	1	0.067	1 ==> 2
448 (m 61: Lower m3 mesiobuc)	2	0.128	2 --> 0
470 (m 83: Lower m1-2 distob)	1	0.136	1 ==> 0

518 (Cranial 33: Epitympanic)	1	0.500	0 ==> 1
623 (Cranial 138: Palate fen)	1	0.250	0 ==> 1
697 (Vertebral 10: Length of)	1	0.111	1 --> 0
715 (Scapular 2: Coracoid pr)	1	0.333	2 --> 1
717 (Scapular 4: craniolater)	1	0.143	0 --> 1
718 (Scapular 5: Infraspinou)	1	0.667	1 --> 2
726 (Scapular 13: Axilloglen)	1	0.235	1 --> 0
733 (Scapular 20: Triceps pi)	1	0.222	0 --> 1
737 (Humeral 3: Capitulum sh)	1	0.154	1 --> 0
738 (Humeral 4: Capitular ta)	1	0.111	1 --> 2
741 (Humeral 7: Medial edge)	1	0.133	0 --> 1
746 (Humeral 12: On the dist)	1	0.071	0 --> 1
747 (Humeral 13: Relative he)	1	0.176	0 --> 1
757 (Humeral 23: Brachialis)	1	0.091	1 --> 0
765 (Humeral 31: Deltopector)	1	0.125	1 --> 2
768 (Ulnar 1: Length of the)	1	0.176	1 --> 0
782 (Radial 1: Shape of the)	1	0.333	1 --> 0
797 (Carpal 7: Lunate fused)	1	0.333	0 --> 1
816 (Innominate 4: Concavene)	1	0.167	1 --> 0
818 (Innominate 6: Anterior)	1	0.211	1 --> 0
820 (Innominate 8: length of)	1	0.192	1 --> 0
824 (Innominate 12: Shape of)	1	0.167	1 --> 0
829 (Innominate 17: Anterior)	1	0.167	0 --> 1
838 (Femoral 4: Length index)	1	0.125	1 --> 2
840 (Femoral 6: Proximal ext)	1	0.318	1 --> 2
861 (Femoral 27: Proximodist)	1	0.143	0 --> 1
868 (Tibial 7: Rotation of t)	1	0.222	1 --> 2
883 (Astragalar 1: Height of)	1	0.087	1 --> 0
886 (Astragalar 4: Trochlear)	1	0.077	1 --> 0
889 (Astragalar 6: Position)	1	0.667	1 --> 2
895 (Astragalar 12: Dorsal e)	1	0.125	1 --> 0
916 (Calcaneal 14: Calcaneoc)	1	0.231	1 --> 0
939 (Cuneiform 7: Prehallux)	1	0.500	1 --> 0
957 (Metatarsal 2 : From pro)	1	0.250	0 --> 1
974 (Metatarsal 19: Stylar p)	1	0.167	0 --> 1
975 (Metatarsal 20: Groove o)	1	0.333	0 --> 1

Table S7.

Synapomorphic characters supporting the Scandentia clade

Characters	Steps	CI	Changes
23 (I 23: Upper I2 size rela)	1	0.136	2 ==> 1
30 (I 29: Upper I3 evolution)	1	0.143	0 ==> 1
34 (I 33: Diastema between u)	1	0.077	1 ==> 2
39 (C 4: Upper canine premol)	1	0.091	0 --> 1
43 (C 8: Mesial crista on up)	1	0.048	0 ==> 1
51 (P 1: Upper P1 presence)	1	0.059	0 ==> 1
56 (P 6: Upper P2 buccolingu)	1	0.091	0 ==> 1
134 (P 81: Upper P4 stylar s)	1	0.182	0 --> 1
167 (M 1: Upper M1 size rela)	1	0.068	0 --> 2
193 (M 25: Upper M2 metaconu)	1	0.239	1 --> 2
218 (M 50: Upper M1-2 metast)	1	0.500	0 ==> 1
225 (M 57: Upper M1-2 stylar)	2	0.120	0 --> 2
230 (M 62: Upper M1-2 distal)	1	0.400	0 --> 1
237 (M 68: Upper M1-2 postpr)	1	0.036	0 --> 1
247 (M 78: Upper M1-2 connec)	1	0.143	1 --> 2
260 (i 4: Lower i2 size)	1	0.074	1 ==> 2
275 (i 19: Lower i2 crown sh)	1	0.444	0 ==> 1
276 (i 20: Lower i2 crown or)	1	0.105	1 ==> 0
286 (c 2: Lower canine size)	1	0.056	0 --> 1
288 (c 4: Lower canine lingu)	1	0.160	0 --> 1
291 (c 7: Lower canine dista)	1	0.111	0 --> 1
294 (c 10: Lower canine prem)	1	0.091	0 --> 1
298 (p 1: Lower p1 evolutioa)	1	0.053	0 ==> 1
328 (p 31: Lower p3 distal m)	1	0.050	0 ==> 1
371 (p 74: Lower p4 entoconi)	2	0.087	0 --> 2
380 (p 83: Lower p4 exodaeno)	1	0.118	0 --> 1
385 (p 88: Lower premolar ov)	1	0.192	1 --> 2
386 (m 1: Lower m1 overlap p)	1	0.125	1 --> 2
421 (m 34: Lower m2 paraconi)	1	0.185	0 --> 1
458 (m 71: Lower m1-2 post-e)	1	0.105	0 --> 1
465 (m 78: Lower m1-2 posten)	1	0.119	1 --> 0
466 (m 79: Lower m1-2 hypocr)	1	0.108	2 ==> 3
468 (m 81: Lower m1-2 waiste)	1	0.050	0 --> 1
471 (m 84: Lower m1-2 extens)	1	0.042	1 --> 0
475 (m 88: Lower m2-3 trigon)	1	0.077	1 ==> 0
482 (m 95: Lower m1-3 pre-en)	1	0.095	1 --> 2
483 (m 96: Lower m1-3 taloni)	1	0.105	1 --> 2
521 (Cranial 36: Pyramidal e)	1	0.143	1 ==> 0
528 (Cranial 43: Ectotympani)	1	0.333	1 ==> 0
533 (Cranial 48: Double prom)	1	0.100	0 ==> 1
548 (Cranial 63: Medial pter)	1	0.333	1 --> 2

549 (Cranial 64: Temporomand)	1	0.200	1 ==> 0
573 (Cranial 88: Medial orbi)	1	0.600	1 ==> 0
586 (Cranial 101: Position o)	1	0.118	0 ==> 1
589 (Cranial 104: Metopic su)	1	0.286	0 ==> 1
591 (Cranial 106: Interparie)	1	0.143	1 ==> 2
599 (Cranial 114: Zygomatic)	1	1.000	0 ==> 1
615 (Cranial 130: Orientatio)	1	0.182	0 --> 1
624 (Cranial 139: Postpalati)	1	0.172	1 ==> 0
632 (Cranial 147: Maxillary)	1	0.091	0 ==> 1
646 (Cranial 161: Tentorium)	1	0.222	1 ==> 2
648 (Cranial 163: Projection)	1	0.105	1 ==> 2
688 (Vertebral 1: C3-7 artic)	1	0.667	0 ==> 2
693 (Vertebral 6: C3-5 ventr)	1	0.100	0 --> 1
694 (Vertebral 7: Expansion)	1	0.250	1 --> 2
711 (Vertebral 24: Vertebra)	2	0.227	1 --> 3
724 (Scapular 11: Acomion pr)	1	0.125	0 --> 1
760 (Humeral 26: Olecranon f)	1	0.167	1 ==> 0
769 (Ulnar 2: Ventral projec)	1	0.125	0 --> 1
777 (Ulnar 10: Crest for m.)	1	0.083	0 ==> 1
811 (Carpal 21: Orientation)	1	0.200	0 ==> 1
846 (Femoral 12: Crista para)	1	0.087	0 ==> 1
869 (Tibial 8: Shape of medi)	1	0.286	1 --> 2
870 (Tibial 9: Shape of dist)	1	0.133	0 --> 1
891 (Astragalar 8: Medial ta)	1	0.400	0 --> 1
899 (Astragalar 16: From dis)	1	0.125	0 ==> 1
941 (Cuneiform 9: Plantar pr)	1	0.167	1 ==> 2
956 (Metatasral 1 : Proximal)	1	0.600	0 ==> 1
1015 (Soft tissular 19: Feta)	1	1.000	2 ==> 1
1024 (Soft tissular 28: Feta)	1	0.667	0 ==> 2
1030 (Soft tissular 34: Feta)	1	1.000	0 ==> 1
1031 (Soft tissular 35: Feta)	1	0.667	1 --> 2
1039 (Diogo&Wood2011_Muscula)	1	1.000	0 --> 1
1044 (Diogo&Wood2011_Muscula)	1	0.500	0 --> 1
1062 (Diogo&Wood2011_Muscula)	1	1.000	0 --> 1
1080 (Diogo&Wood2011_Muscula)	1	1.000	0 --> 1
1090 (Diogo&Wood2011_Muscula)	1	0.333	0 --> 1
1099 (Diogo&Wood2011_Muscula)	1	1.000	0 --> 1
1106 (Diogo&Wood2011_Muscula)	1	1.000	0 --> 1
1120 (Diogo&Wood2011_Muscula)	1	1.000	0 --> 1
1122 (Diogo&Wood2011_Muscula)	1	0.500	0 --> 1
1123 (Diogo&Wood2011_Muscula)	1	1.000	0 --> 1
1154 (Diogo&Wood2011_Muscula)	1	0.500	0 --> 1
1196 (Diogo&Wood2011_Muscula)	1	0.500	0 --> 1
1201 (Molecular 2: SINEs and)	1	1.000	0 ==> 1
1202 (Molecular 3: SINEs and)	1	1.000	0 ==> 1

Supplementary Datasets as TNT and NEXUS files (separate files)

Ni_2015_Treeshrews_TNT.doc
Ni_2015_Treeshrews_NEXUS.doc
